# Sturge–Weber syndrome: updates in translational neurology

**DOI:** 10.3389/fneur.2024.1493873

**Published:** 2024-12-02

**Authors:** Chase Solomon, Anne Comi

**Affiliations:** ^1^Neurology and Developmental Medicine, Kennedy Krieger Institute, Baltimore, MD, United States; ^2^Department of Neurology, Johns Hopkins School of Medicine, Baltimore, MD, United States; ^3^Department of Pediatrics, Johns Hopkins School of Medicine, Baltimore, MD, United States

**Keywords:** Sturge–Weber syndrome, models, blood brain barrier, treatment, seizure, diagnosis

## Abstract

Sturge–Weber syndrome (SWS) is a rare congenital neurovascular disorder that initially presents with a facial port-wine birthmark (PWB) and most commonly associated with a R183Q somatic mosaic mutation in the gene *GNAQ*. This mutation is enriched in endothelial cells. Contrast-enhanced magnetic resonance imaging (MRI) diagnoses brain abnormalities including leptomeningeal vascular malformation, an enlarged choroid plexus, and abnormal cortical and subcortical blood vessels. Mouse SWS models identify dysregulated proteins important for abnormal vasculogenesis and blood brain barrier permeability. Recent clinical research has focused on early diagnosis, biomarker development, presymptomatic treatment, and development of novel treatment strategies. Prospective pilot clinical drug trials with cannabidiol (Epidiolex) or with sirolimus, an mTOR inhibitor, indicate possible reductions in seizure frequency and improved cognitive outcome. This review connects the most recent molecular research in SWS cell culture and animal models to developing new treatment methods and identifies future areas of research.

## 1 Introduction

Sturge–Weber syndrome (SWS) is a rare neurovascular disorder present at birth that is characterized by a facial port-wine birthmark (PWB), abnormal blood vessels in the brain and eyes, including leptomeningeal involvement. Historically, it is categorized with other phakomatoses; disorders in this classification include neurofibromatosis 1, tuberous sclerosis complex, and von Hippel–Lindau Disease (1). These disorders are characterized by cutaneous lesions, neuro-ophthalmic defects, and tumor formation (2, 3); however, it is now known that SWS is pathophysiologically more like other genetic neurovascular disorders, including Cerebral Cavernous Malformation and Hereditary Hemorrhagic Telangiectasia, except that bleeding is not a prominent part of the presentation at least in children. In addition, SWS is not hereditary and rather occurs sporadically during fetal development. The estimated incidence of SWS is 1 in 20,000 to 50,000 live births, with more recent population studies obtaining incidence rates of 0.19 per 100,000 people per year and 3.08 per 100,000 people per year (4, 5). Typical brain abnormalities of SWS patients include cortical and sub-cortical peri-vascular calcification, impaired venous drainage and perfusion, and brain atrophy of varying degree; tumors are not common. Individuals may present with seizures and stroke or stroke-like episodes; as a result, they can experience both cognitive and neurodevelopmental deficits.

SWS is caused primarily by a c.548G➔A (p. Arg183Gln) somatic mosaic mutation in *GNAQ*, hypothesized to be enriched in endothelial cells (6–8). *GNAQ* codes for the protein Gαq; when expressed in mutant form, Gαq demonstrates impaired deactivation resulting in hyperactivation of downstream pathways, including the Ras–Raf–MEK–ERK (also known as the MAPK/ERK pathway) and the mammalian target of rapamycin (mTOR) pathways (9, 10). Human SWS brain tissue immunohistochemistry has indicated increased expression of phosphorylated ERK (p-ERK) and decreased expression of CD34 in endothelial cells from abnormal blood vessels in the leptomeninges (11). The presence of the R183Q *GNAQ* mutation in abnormal scleral tissue correlated with increased expression of p-ERK and p-JNK in endothelial cells that line blood vessels (12). When comparing lesioned brain tissue from SWS patients to epilepsy controls, researchers noted a greater likelihood of phosphorylated-S6 staining in the leptomeningeal endothelial cell layer of SWS brain tissue (13). Fibroblasts derived from SWS port-wine birthmark skin showed significantly higher levels of fibronectin gene expression compared to SWS normal skin (14). The R183Q mutation in *GNAQ* has also been identified as the primary mutation within blood vessels of PWBs (15). Upregulated vascular endothelial growth factor (VEGF)-A and VEGF receptor 2 (VEGFR2) are both found in PWB tissue and may contribute to abnormal MAPK/ERK pathway regulation (10, 16). Abnormal protein expression in human tissue and *in vitro* studies suggest that these pathways are targets to study with *in vivo* models and in clinical trials. Mouse models, using the R183Q mutation in *GNAQ,* have recently been developed to investigate abnormal molecular and vascular features of SWS and to promote preclinical drug and gene therapy research. The aim of this review is to highlight the clinical and genetic background of Sturge–Weber syndrome, provide an update on recent advances related to Sturge–Weber syndrome, and identify current gaps and potential future developments in molecular and clinical research.

## 2 Signs and symptoms

**Figure 1:**
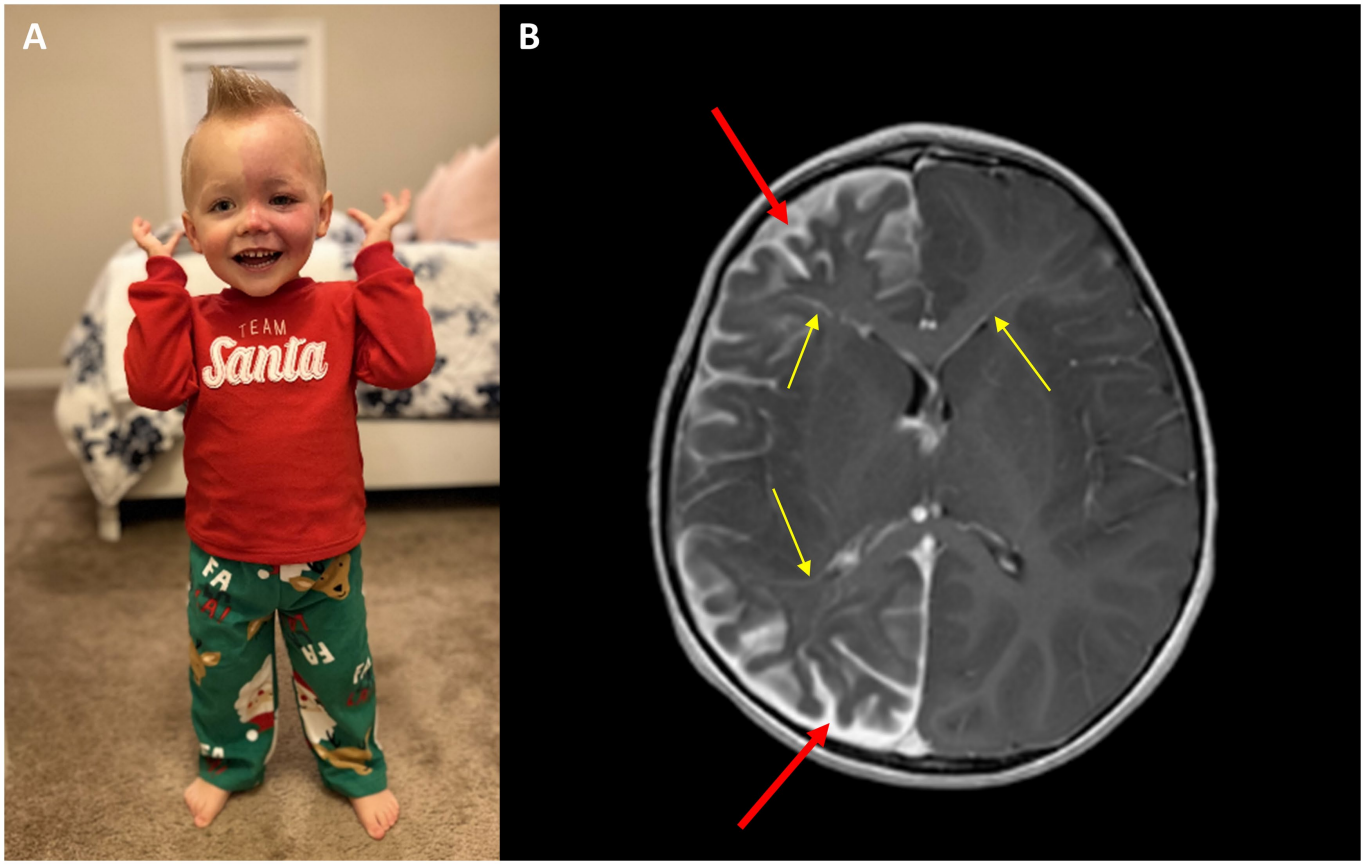
**(A)** Image of a SWS patient with a left facial port-wine birthmark and **(B)** corresponding T1 post-contrast MRI indicating leptomeningeal involvement and abnormal blood vessels, indicated by red arrows. Yellow arrows indicate enlarged deep veins that flow into the cortex.

PWBs are commonly found in about 3 in 1,000 births. Only 6% of babies born with a facial PWB also have brain involvement and develop the common neurological deficits of SWS. A PWB on the forehead, temple region or upper eyelid yields a risk of 20 to 50% for disease brain involvement, and when a PWB covers both the upper and lower eyelid, the risk of eye involvement and glaucoma is 50% (17). Figure 1A depicts the typical phenotype of a high risk facial PWB. Figure 1B shows the patient’s corresponding T1 post-contrast MRI image, which shows typical SWS leptomeningeal involvement and abnormal blood vessels. Previous research has established a positive correlation between the size of a facial PWB, the degree of SWS brain involvement, and severity of SWS-related neurological outcomes (18). Facial PWB size may be used as a predictor of the extent of neurological disability for an individual with SWS. Analyses of pattern distribution of a facial PWB are useful in determining SWS diagnosis. Commonly seen patterns of PWB include linear, frontotemporal, isolated cheek and canthus, combined linear and cheek, hemifacial, and median; hemifacial and median patterns are strongly associated with an increased risk of SWS (19, 20). Facial PWB often respond well to laser treatment and can safely be initiated in young infants (21).

Elevated eye pressure and glaucoma is seen in 30 to 70% of patients with a PWB that covers both the upper and lower eyelid. Glaucoma can present at birth with eye enlargement (bupthalmus), however increased eye pressure may also develop in infancy or later into adolescence. Because of this, it is suggested that an ophthalmological exam be performed as soon as possible, and that patients be regularly monitored for life. Untreated glaucoma can ultimately result in ischemic ocular injuries and permanent vision loss (22). Treatment includes eye drops, which lower eye pressure, and when unsuccessful, various surgical techniques may be implemented to accomplish this (23–25). Thickening of the choroid and retinal detachment are complications which occur in a subset of patients; treatment of retinal detachment can help recover vision in some of these patients (26).

The most common presenting neurologic symptom for children with SWS are seizures, which are exhibited in 75% of children with SWS within 1 year post-birth and 90% within 2 years post-birth (27). Seizures, in babies and young children, can further impair blood flow to the brain and can accelerate neurological deterioration. Patients with bilateral brain involvement are more susceptible to seizures than patients with unilateral involvement; the age of onset in patients with bilateral involvement also tends to be earlier than those with unilateral involvement (28). 75% of patients with unilateral involvement experience seizures; this increases to 95% for patients with bilateral involvement (28, 29). Seizures are most commonly focal motor seizures with or without impaired consciousness; less commonly seen seizure types include myoclonic epilepsy or infantile spasms (30, 31). It is important for a parent or guardian to be educated on typical seizure semiology in order to recognize the possible ways these children can present with seizures.

Seizures can trigger other symptoms such as stroke-like episodes and migraines. Conversely, migraines have the potential to trigger seizures and stroke-like episodes (22, 32). Status epilepticus is a classification of seizure which either lasts for at least 5 minutes or involves multiple seizures without returning to a base level of consciousness between episodes. It is common for SWS patients and is associated with stroke-like episodes, which can be defined as transient unilateral weakness with or without prior seizure activity (33). Stroke-like episodes may be accompanied by resulting neurologic deficits; these can also be acquired over time without a stroke-like episode or seizure as a trigger (34). Because toddlers and children with SWS are more susceptible to stroke-like episodes from minor head injury, it is advisable for children to avoid contact-heavy recreational activities that involve constant physical use or involvement of the head (22).

Cognitive impairments and deficits are common in SWS patients; these can include learning disabilities, attention problems, or other behavioral deficits. Bilateral brain involvement and an earlier age of onset of seizures both potentially contribute towards severe cognitive impairment (29). One retrospective study suggests that the combination of stroke-like episodes and seizures in SWS patients is a driving factor behind the development of hemiparesis and intellectual disorders as well as an increased risk in developing drug-refractory epilepsy, or DRE (35). See Mesraoua et al. for a comprehensive review on DRE, which is first identified in a patient when they are given two separates, tolerated antiepileptic drug schedules and fail to experience seizure control (36). While DRE presents increasing medical challenges for SWS patients, it has been shown to be attenuated through more aggressive initial therapy (37).

## 3 Genetics

In 2013, cause of SWS was discovered to be a single nonsynonymous mutation: the c.548G → A (p.Arg183Gln) mutation in the gene *GNAQ* present in roughly 90% of patients (6) in affected brain, skin and eye tissue. The R183Q mutation has also been reported to be enriched in endothelial cells (8). It is hypothesized that this somatic mutation occurs during the early stages of embryonic development (38); however, the exact timing in which the mutation occurs in fetal development may vary and mutations that occur later on into development will have a local effect. Mutations that occur early on likely involve more cell types and have a greater chance to cause disease brain involvement (39).

**Figure 2:**
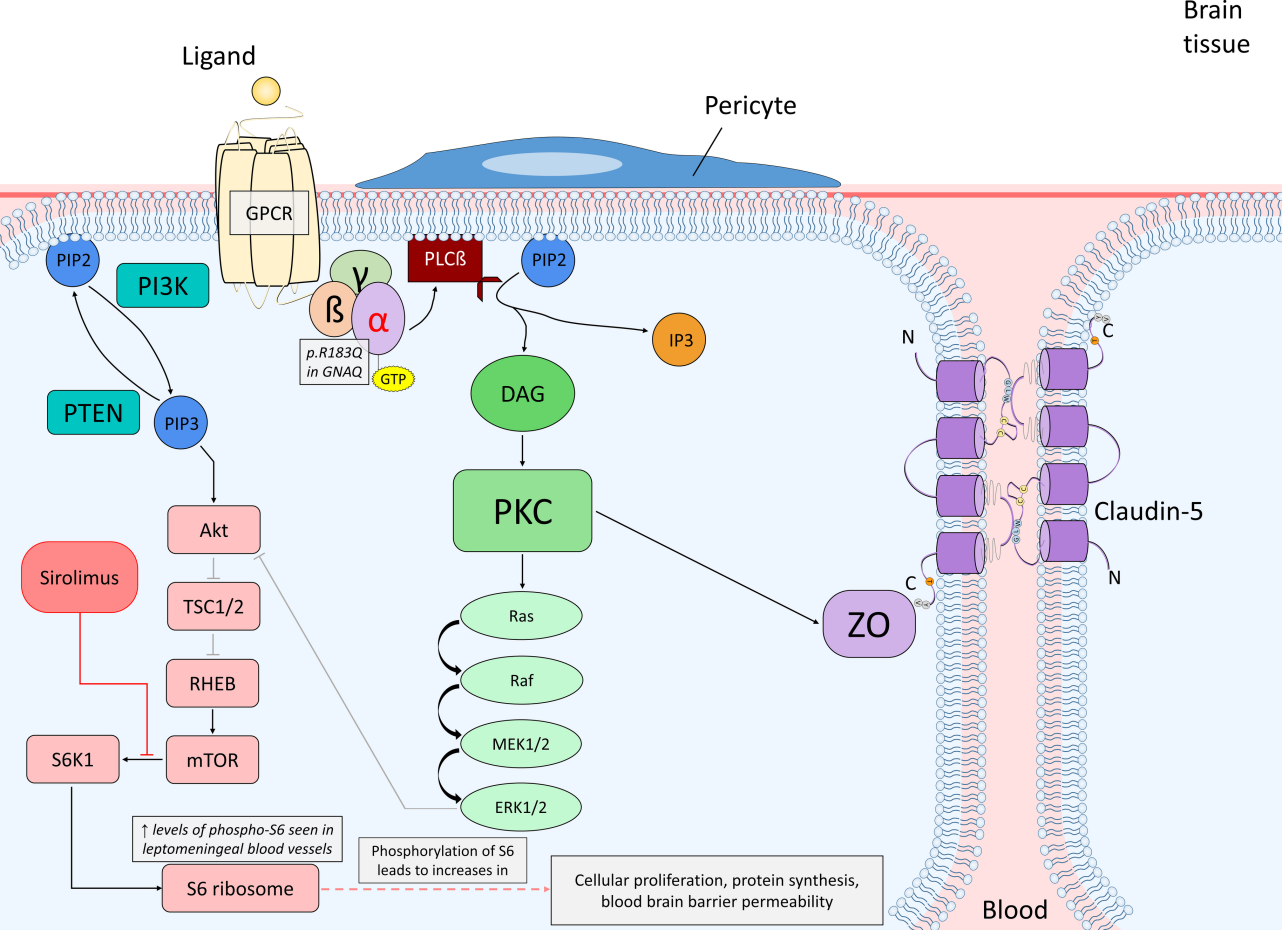
Diagram of endothelial cell mutant Gαq signaling (p.R183Q in *GNAQ*) and the primary affected downstream pathways, the Ras–Raf–MEK–ERK and mTOR pathways, within the context of the blood brain barrier. The alpha subunit becomes active when bound to GTP, which promotes PLCß to cleave PIP_2_ into DAG and IP_3_. DAG is able to activate PKC, which can then phosphorylate a variety of proteins. In this case, PKC can initiate the activation of the Ras–Raf–MEK–ERK pathway or phosphorylate ZO, which interacts with claudin-5 to maintain the integrity of endothelial tight junctions. ERK may stimulate cellular proliferation or also play a role in PI3K/Akt inhibition. PIP_2_ may also be converted into PIP_3_ by PI3K; PTEN dephosphorylates PIP_3_ back to PIP_2_. PIP_3_ activates the Akt signaling pathway once bound to Akt, which can inhibit TSC1/2 via phosphorylation. TSC1/2 are negative regulators of mTOR due to their inhibitory mechanism on RHEB, an activator of mTOR. The inhibition of TSC1/2 by Akt allows for RHEB to activate mTOR. Proper expression of mTOR activates S6K1, which then phosphorylates the 40S ribosomal subunit S6 protein (phosphorylated-S6). Increased levels of phosphorylated-S6 result in increased cellular proliferation, protein synthesis and blood brain barrier permeability. Sirolimus acts as an inhibitor of mTOR by causing rapid inactivation of S6K1, and as a result, preventing phosphorylation of the S6 protein and further cellular proliferation as well as vascular growth. A. Abbreviations: phospholipase C ß (PLCß); phosphatidylinositol 3,4,5-bisphophate (PIP_2_); diacylglycerol (DAG); inositol 1,4,5-trisphosphoate (IP_3_); protein kinase C (PKC); zonula occludens (ZO); phosphatidylinositol 3,4,5-trisphophate (PIP_3_); phosphoinositide 3-kinase (PI3K); phosphatase and tensin homolog (PTEN); tuberous sclerosis proteins 1 and 2 (TSC1/2); Ras homolog enriched in brain (RHEB); p70 ribosomal S6 kinase (S6K1).

*GNAQ* codes for the protein Gαq, which is a component of the trimeric G protein complex and associates with G protein coupled receptors (GPCRs). GPCRs associated with Gαq include glutamate, histamine, angiotensin, and vasopressin receptors and generally impact cellular processes affecting protein activity and phosphorylation regulating cellular proliferation and differentiation (40–43). The R183Q mutation in *GNAQ* results in the hyperactivation of Gαq due to impaired auto-hydrolysis, and thus a decreased rate of dissociation of Gαq from GTP and re-association with GDP. In the case of R183Q in *GNAQ*, hyperactive Gαq leads to hyperactivation of downstream pathways in endothelial cells, resulting in capillary malformations (7). Downstream hyperactive pathways such as the Ras–Raf–MEK–ERK pathway and the mTOR pathway are shown in Figure 2. The abnormal regulation of such pathways and their role in vascular malformation in SWS is highlighted in previous studies and a focus of studies currently taking place (8, 11).

While the R183Q *GNAQ* mutation is found most commonly in SWS patients, other somatic and germ line mutations have been reported to induce SWS brain involvement. *GNA11* is a paralogue gene of *GNAQ*; mutations of *GNA11* have been reported in individuals with SWS in two separate studies (44, 45). Hyperpigmentation is commonly seen in *GNA11-*related SWS cases, while SWS patients with the *GNAQ* mutation, are more likely to experience significant hemispheric brain atrophy and have seizures (46). The aforementioned differences between individuals with SWS with the R183Q *GNAQ* and the R183C *GNA11* mutation are important to consider in developing future treatment applications. Another study reported a novel somatic mutation (p.K78E) in the gene *GNB2*, which encodes for the *β* subunit of the G-protein complex in a skin biopsy from a patient with SWS. The mutation, which occurs via the substitution of lysine to glutamic acid results in the loss of the cationic ammonium from Lys78 and disrupts the salt bridge. The loss of an ammonium creates a charge repulsion and impairs the binding affinity of the *β* subunit to the *α* subunit. Functional studies with cells with the *GNB2* mutation demonstrated no downstream effect on MAPK signaling. However, both the *GNB2* and R183Q *GNAQ* mutation have direct influence on Yes-associated protein (YAP) expression, as endothelial cell YAP is reduced in both cases (47). The similarities in YAP expression between the *GNB2* and *GNAQ* mutation suggest that YAP expression levels through the Hippo pathway may play a role in the pathogenesis of SWS, although further evidence of this is needed.

Recent research suggests that genetic testing should be performed when atypical features are present in an individual with a facial port-wine birthmark (48). Other somatic variants in individuals with atypical features of SWS include G48V in *GNAQ*, R183C in *GNA11*, M1043I in *PIK3CA*, and a mosaic deletion involving *PTPRD* and *PTPRD-AS2*. Germ line mutations were detected in the *RASA1*, *EPHB4*, and *KIT* genes of patients with atypical SWS features. *RASA1* and *EPHB4* germ line mutations are generally associated with capillary malformation-arteriovenous malformation syndrome, a disorder that increases the risk of fast-flow malformations and pleural effusion as well as other lymphatic anomalies (49, 50). Patients with reported mutations in either *RASA1* or *EPHB4* had family histories of capillary malformation. It is important to consider other genetic causes when atypical phenotypes are seen in skin presentation, MRI abnormalities, family history, or in other symptoms and features that deviate from typical SWS phenotype. Work is ongoing to better understand genotype–phenotype associations.

## 4 Updates on models for SWS

Accurate representation of SWS through models is necessary to continuously elucidate the impact of the R183Q *GNAQ* mutation on vascular development and for testing of new therapies and drug targets in the pre-clinical setting. It is important to differentiate models with the Q209L *GNAQ* mutation (51–53) from models with the R183Q *GNAQ* mutation (54, 55). While both are understood to be hyperactivating mutations, the Q209L mutation results in much greater hyperactivation (56, 57). The Q209L *GNAQ* mutation has never been reported with SWS or facial PWB; rather the Q209L mutation has been associated with vascular tumors (58–60). The PWBs seen in SWS are vascular malformations rather than vascular tumors (61, 62).

### 4.1 R183Q GNAQ *in vitro* models

Modeling the R183Q *GNAQ* mutation *in vitro* began initially with transient transfection of cells and has progressed to stable transfection of cells from mice or humans, obtained from tissue, and studied continuously through cell culture (6, 47, 52). Multiple models isolating endothelial cells for variants in *GNAQ* mutations have been generated recently. Skin samples from mice injected with mutant R183Q GNAQ endothelial cells combined with bone marrow mesenchymal progenitor cells had a greater percentage of enlarged vessels compared to skin samples injected with wild type endothelial cells (63). The R183Q *GNAQ* mutation, when expressed in endothelial cells, resulted in constitutive activation of phospholipase Cβ (PLCβ), which plays a major role in cell signaling through the G-protein cycle. PLCβ generates other active molecules such as inositol 1,4,5-triphosphate (IP_3_) and diacylglycerol (DAG). DAG, in particular, activates protein kinase C, which when activated, phosphorylates various proteins that can regulate membrane permeability, cellular proliferation, and control metabolic pathways. Angiopoietin-2 (Ang2) levels were increased in the abnormally large blood vessels of mice injected with mutant endothelial cells, and when treated with higher concentrations of AEB071, an inhibitor of PKC, levels of Ang2 were reduced to a normal, non-SWS phenotypic level (63), suggesting that constitutive activation of PLCβ plays a role in the phenotype of enlarged blood vessels.

A recent brain tissue study in 4 samples from patients with SWS indicated the presence of cells with multiple macrophage-associated molecules such as MRC1, CD163, CD68, and LYVE1 that were absent in the human control brain tissue from 2 patients. ICAM1, an endothelial cell-specific protein that promotes leukocyte adhesion and regulates cellular responses in inflammation, was also expressed at higher levels in the human SWS brain tissue (64). ICAM1 was also identified in the endothelial layer of some blood vessels. This work suggests that macrophages are potentially recruited to leaky perivascular areas where ICAM1 is expressed and play a role in the regulation of angiogenesis.

A common outcome of SWS seen in patients is brain calcification, likely due to the interactions of venous hypertension, brain ischemia, and seizures that worsen blood flow, with the R183Q *GNAQ* mutation in impacted cells. When IP_3_ is produced from phosphatidylinositol 4,5-bisphosphate (PIP_2_) being cleaved by PLCβ, it triggers an influx of calcium from the endoplasmic reticulum into the cell. Mutant R183Q HEK293 cells show increased production levels of IP_3_ than wild-type cells, suggesting that the mutation results in a greater influx of calcium in the cytoplasm (65). Hyperactivated calcium signaling has been identified in mutant R183Q telomerase-immortalized microvascular endothelial (TIME) cells, accompanied by increased inositol monophosphate, which indicates the presence of IP_3_ before initiating the release of calcium into the cell and degrading (66). This however, was attenuated by inhibition of the calcium-release-activated channel. While inhibition in this case does not reverse previous calcification found in blood vessels, the dynamic of regulating calcium within cells is worth understanding further in the context of mutant Gαq and its affected pathways.

### 4.2 R183Q GNAQ *in vivo* models

A mouse model using cre-drivers to express the R183Q *GNAQ* mutation during embryonic development was created to investigate the impact of the mutation in different cell types during fetal development (54). In *Gnaq*RQ^wt/wt^;*β*-actin-Cre + x *Gnaq*RQ^fl/wt^ transgenic mice, which express the R183Q *GNAQ* mutation globally, no mice were born containing both the Cre driver and the conditional *GNAQ* allele, suggesting that the mutation results in complete embryonic lethality when expressed during very early stages of development. When the R183Q *GNAQ* mutation was expressed in endothelial cells in a mosaic manner (mutation is present in only some cells), the transgenic mice that survived after birth did not exhibit any notable vascular defects.

A tetO-GNAQ*R183Q X *Tie2-rtTA/TRE-βGal* (R183Q *GNAQ* mice) transgenic mouse model was recently created using doxycycline induced expression of the mutation in endothelial cells in mutant mice at age P15 (55). When perfused following injections with Evans Blue dye, a significantly higher percentage of mutant mice brains had severe Evans Blue staining in their brain; no littermate control mice exhibited severe Evans Blue staining. Phosphorylated-S6, which is an indicator of increased mTOR activity (67), was found at increased expression levels in leptomeningeal blood vessels of mutant mice, compared to littermate control mice, a finding similar to that reported in human brain tissue (13). Microvessels in the region of the retrosplenial cortex showed irregular and discontinuous expression patterns of both phosphorylated-S6 and claudin-5. Claudin-5 is a tight junction protein that contributes significantly to the integrity of the blood brain barrier (68). These results suggest that the blood brain barrier of mutant, R183Q *GNAQ* mice are significantly more permeable than that of their littermate controls, and that mTOR is likely to be found at elevated levels in the leptomeninges, suggesting involvement of this pathway as well. Other studies link mTOR inhibition and PI3K/Akt pathway inhibition to restoring blood brain barrier integrity through increased expression of tight junction proteins and decreased autophagy (69, 70). This suggests that mTOR inhibition may act in a similar manner for SWS, in which models already indicate increased phosphorylated-S6 and a compromised blood brain barrier (13, 55).

Angiopoietin-2, which acts as an antagonist towards angiopoietin-1, promotes vessel instability and leakiness by causing pericyte detachment (71). Ang2 plays a large role in the permeability of the blood brain barrier during angiogenesis (63, 72, 73). Together, the recent data from the mouse model and human tissue to date supports that the R183Q mutation in *GNAQ* plays a role in both blood vessel overgrowth via constitutive activation of PLCβ3, which results in irregular downstream pathway activity in the MAPK/ERK and mTOR pathways, resulting in abnormally high Ang2 expression, and breakdown of the blood brain barrier (see Figure 2) as well as abnormal macrophage invasion of the involved cortex.

## 5 Advances in outcome measurements

The SWS-Neurological Rating Score (SWS-NRS), or Neuroscore, is used as a cumulative assessment of the extent of neurological impairment in SWS patients (74). It is a metric that is comprised based off observed visual defects, seizure frequency, extent of hemiparesis, and degree of cognitive function. SWS Neuroscore has been used in various studies in comparison with MRI imaging and EEG evaluations and may serve as an important measurement when administering a certain treatment or therapy (75–77). The NIH Quality of Life in Neurological Disorders (Neuro-QoL) measures the physical, mental, and social effects of neurological conditions in both children and adults. The extent of skin, total eyelid port-wine birthmark, eye, and overall SWS involvement were negatively correlated with cognitive function Neuro-QoL; as the involvement of SWS becomes more diffuse, patients typically see a drop in quality of life (78). Previous research has also shed light on linking SWS to increased suicidality compared to other neurological disorders (79). Continued research is necessary to understand whether this is due the disease pathology of SWS, facial port-wine birthmark, epilepsy or other factors.

### 5.1 Updates in treatment

#### 5.1.1 Low dose aspirin

Multicenter studies for SWS have recently been conducted on individuals with SWS to understand current treatment patterns (77, 80). Studies show that low-dose aspirin is often recommended and used for management of seizures and stroke-like episodes (81, 82). However, adverse effects to aspirin for Sturge–Weber patients have been reported previously as well (83, 84). Lance et al. reports other side effects from SWS patients taking aspirin, however it is notable that only a small percentage of SWS patients with brain involvement in this study experienced side effects (81). It is recommended to treat hemiparetic stroke-like episodes with aspirin, in hopes of abating ischemia due to vascular malformation (85). Aspirin has also been taken along with levetiracetam in patients both pre-symptomatically and after the onset of symptoms (86, 87). When determining whether low-dose aspirin is an option for a SWS patient, one must consider the severity and degree of brain involvement as well as any symptoms that the patient already exhibits.

#### 5.1.2 Presymptomatic treatment

If SWS diagnosis is obtained prior to the onset of symptoms, presymptomatic treatment with aspirin and antiepileptic drugs in low doses may aid in delaying the onset of seizures (84). Screening for brain abnormalities (calcification, blood vessel abnormalities) prior to the onset of symptoms may help in calculating the risk of future brain involvement as well as encourage presymptomatic treatment (88). Early MRI with gadolinium enhancement, when possible, aids in accurate determination of SWS brain involvement and allows for the potential for presymptomatic treatment (89). Recent studies suggest that rapid “feed & wrap” non-contrast MRIs can be obtained in young infants, and show various vascular and parenchymal (indirect) signs of SWS that could be used to detect presymptomatic SWS brain involvement (90). The argument against this, however, presents that in infants, MRI requires anesthesia and can potentially show false-negative results that are more difficult to elucidate early on into postnatal development (91). Conversely, especially for high-risk patients, early detection of SWS brain involvement leading to presymptomatic treatment may be beneficial; this of course should be discussed and implemented on a case-by-case basis. Shortened MRI may be useful and effective in infants that are not sedated (92). Further prospective study of non-sedated, non-contrast MRI is being used by various centers, along with neurological examination and EEG to screen for brain involvement. This strategy may prove useful in future presymptomatic drug trials that would require pre-treatment verification of brain involvement (90). The potential drawbacks of sedation in infants requiring MRI as well as different approaches to mitigate the risks of sedation have been previously described (93). Retrospective analyses show that presymptomatic treatment results in a noticeable delay in age of seizure onset as well as a lower (improved) hemiparesis Neuroscore (94). In this study, it is notable that the presymptomatic treatment group had a higher percentage of both bilateral brain involvement and skin involvement. Delaying the onset of seizures in infants with presymptomatic treatment allows for more normal neurological development with a lower likelihood of future brain injury and cognitive impairment (95).

### 5.2 mTOR inhibition

#### 5.2.1 Mouse studies

Increased expression of phosphorylated-S6, which is a downstream target of the mammalian target of rapamycin (mTOR) pathway, is observed in multiple models with the R183Q *GNAQ* mutation (13, 55, 96). Another animal model that used leukosomes to package rapamycin as a biomimetic drug delivery system showed that mice, when given rapamycin in encapsulated form, showed suppressed endothelial cell proliferation and recovery of normal vessel structure from an inflamed state (97). It is hypothesized that somatic mutations in *GNAQ* that cause hyperactivation of the Ras–Raf–MEK–ERK pathway, and specifically ERK expression, also result in increased activation of mTOR in mutated endothelial cells lining blood vessels. Elevated mTOR pathway activity is commonly known to increase transcription rates, enhance nucleotide, protein, and lipid synthesis, and promote cellular proliferation, which can lead to tumor growth (98). In SWS patients, the increased cellular proliferation is seen through dysregulated angiogenesis primarily in the brain, eye, and facial regions. Patients with SWS, therefore, could potentially receive effective treatment from an mTOR inhibitor.

Growth factors mediate the activation of mTOR and influence rates of protein synthesis. Sirolimus, also known as rapamycin, is able to inhibit the mTOR pathway, and thus protein synthesis, by causing rapid inactivation of S6K1, which is a downstream target of mTOR and essential for the phosphorylation of the ribosomal S6 protein, a protein directly involved in protein synthesis (99, 100). S6K1, when active, facilitates the activation of CREMτ, which belongs to the cAMP-response-element-binding family of transcription factors, and induces further gene transcription and transcription of proliferating cell nuclear antigen (PCNA), which plays an important role in cellular proliferation and DNA synthesis in the S-phase of the cell cycle (101, 102). Through inactivating S6K1, sirolimus is able to inhibit these downstream events. Figure 2 describes the application of sirolimus in mTOR inhibition in the context of G protein-coupled receptor signaling. Previously, sirolimus has been used in multiple mouse models to treat vascular malformation disorders (103, 104). Sirolimus may also minimize the risk of stroke or stroke-like episodes and can potentially better stabilize seizures in mice (105). A clinical trial using topical rapamycin combined with pulsed dye laser (PDL) on the lateral areas of a PWB showed decreased skin pigmentation and a reduced frequency of blood vessels throughout the brain tissue (106).

#### 5.2.2 Human studies

More recently, oral sirolimus has been proposed as another delivery mechanism for SWS patients. In an open-label prospective study with ten subjects with SWS brain involvement and cognitive impairments, patients were given oral sirolimus for 6 months, which resulted in a consensus of improvements in quality of life, cognitive function, and improvements in processing speed (107). The adverse effects of sirolimus for these ten patients were mild, concluding that sirolimus is generally safe for SWS patients and may contribute to benefits in cognitive ability specifically for patients that have previously experienced stroke-like episodes. For a group of patients with DRE, oral sirolimus controlled epileptic symptoms in all patients and resulted in improvements in hypertrophy of pathological tissue (108). Oral sirolimus administered for a SWS patient with left facial hemihypertrophy resulted in depigmentation of their port-wine birthmark as well as decreased soft tissue overgrowth (109). Notably, PDL was not used for this patient. Another case with diffuse choroidal hemangioma has been reported to be treated with oral sirolimus as an adjuvant therapy for PDL of the port-wine birthmark (110). There is a wider range of clinical application of mTOR inhibition in other mTOR pathway-linked diseases, especially tuberous sclerosis (111). A phase III trial of sirolimus for vascular malformations also indicates that side effects such as stomatitis are clinically manageable (112). More extensive research on the application of sirolimus for SWS patients is necessary, especially when treating patients with histories of stroke and stroke-like episodes.

### 5.3 Cannabidiol

#### 5.3.1 Mouse studies

Cannabidiol (CBD) has emerged as a novel therapeutic for many facets of health such as anxiety, depression, insomnia, PTSD, and schizophrenia amongst other diseases. There is growing literature on CBD and its potential application and benefits regarding epilepsy and seizure disorders (113–115). In order to make the case for preclinical drug trials with CBD, animal models must be established proving clear improvements in seizure activity. Two models using CBD to attenuate seizure activity as part of Dravet syndrome each achieve seizure reduction by inhibiting GPR55 (116, 117). CBD has also produced anti-inflammatory effects in a mouse model for Parkinson’s that also suggests GPR55 as a viable target (118). GPR55 is a G protein-coupled receptor that functions as a cannabinoid receptor and is involved in regulating blood pressure as well as cytoskeletal modulation (119). Future clinical work may target GPR55 and other receptors associated with epilepsy and seizure reduction. Multiple mouse models for epilepsy have been produced, further demonstrating the beneficial effects CBD has on seizure reduction and prevention as well as improving social behavior (120–122). In another model looking at chronic CBD treatment, there was no apparent change to the frequency of seizures, and no delay to the onset of seizures (123). There may be differences in outcome between short-term versus chronic administration of CBD that have yet to be explored. In mice given kainate injections to induce seizures, pharmaceutical CBD at the highest dose of 240 mg/kg significantly decreased the severity and frequency of seizures, whereas chronic administration of artisanal CBD did not reduce seizure severity to the same degree as pharmaceutical CBD (124). CBD has yet to be applied to a mouse model specifically for SWS and could potentially be explored in the future.

#### 5.3.2 Human studies

In 2018, the FDA approved Epidiolex, also known as cannabidiol (CBD), for the treatment of seizures for two separate pediatric disorders, Lennox–Gastaut syndrome and Dravet syndrome, which both involve epilepsy (125). Since then, Epidiolex has expanded towards treatment with other disorders relating to seizures and epilepsy, including SWS. A recent multicenter study which treated patients with epilepsy and myoclonic-atonic seizures as well as SWS patients with CBD allowed for reduced seizure frequency in all patients; over half of all patients saw a seizure reduction of at least 50% (126). Epidiolex has also been used in clinical trials only involving SWS patients. The first, which aimed to abate seizure intensity as well as reduce the frequency of seizures in SWS patients, resulted in improved quality of life, significant seizure reduction, and other improvements such as cognitive function, speech and communication, and physical capability (127). Another clinical trial with Epidiolex for ten SWS patients reported no seizures after 6 months of administering oral CBD (128). This was also accompanied by noticeable improvements in SWS Neuroscore and patient-reported quality of life. CBD is suggested to have neuroprotective abilities by inhibiting the mTOR pathway indirectly through JNK inhibition (129). It is a cannabinoid without the psychoactive properties, and administration has proven to be safe and effective for patients with treatment-resistant epilepsy (126, 130). Further investigation into CBD use for SWS patients as well as the potential role that CBD plays in the molecular inhibition of SWS-related abnormally regulated pathways is needed.

## 6 Discussion

Aggressive seizure management has been the mainstay of neurologic management in SWS for the last 25 years. In the last eleven years since the discovery of the p.R183Q mutation in *GNAQ*, the focus of research has shifted towards constructing transgenic animal models, whether *in vivo* or *in vitro*, to further understand the mutation and the downstream pathways and proteins it affects. Through the evidence presented in these models, we can start to implement known inhibitors (such as sirolimus) in prospective pilot trials. The way in which the clinical field has assessed diagnosis and treatment over the past decade has changed as well. MRI with and without contrast has become the gold standard for proper SWS identification. Patients and their families are encouraged to opt for presymptomatic treatment with low-dose aspirin or other anti-epileptic drugs in attempt to delay seizure onset and ameliorate neurocognitive function. Treatment focusing on mTOR inhibition, specifically with sirolimus, has become increasingly more common in the clinical setting. Further research is still necessary regarding the effects of sirolimus on SWS patients and the potential role it may play in management of symptoms for those who experience stroke or stroke-like episodes. Continued research with cannabidiol regarding managing SWS patients’ symptoms as well as its context in an animal model for SWS will be important to reinforce its potential clinical use. It is important that research conducted in the clinical setting runs parallel to that in the bench-laboratory research setting; by modeling SWS through the R183Q mutation in *GNAQ* via cell culture and animal models, we can achieve a more lucid understanding of disease manifestation and progression.
